# Blebs, Barriers, and Bagpipes: Why is It so Hard?

**DOI:** 10.5005/jp-journals-10008-1206

**Published:** 2016-10-29

**Authors:** Michael Coote

**Affiliations:** Associate Professor, Department of Center for Eye Research Australia, The University of Melbourne, Parkville, Victoria, Australia

**Keywords:** Capsule, Glaucoma, Glaucoma device, Outflow, Porosity, Surgery.

## Abstract

Doug Johnson was a clinician-scientist who made great contributions to the understanding of outflow from the eye. This lecture is in honour of Doug and explores the author's understanding of outflow in the surgical context.

**How to cite this article:**

Coote M. Blebs, Barriers, and Bagpipes: Why is It so Hard? J Curr Glaucoma Pract 2016; 10(3):79-84.

## INTRODUCTION

I am greatly honored to be invited to give this lecture. Doug Johnson was a consummate “clinician scientist,” who contributed much to our understanding about outflow of aqueous from the eye. Doug developed a perfused anterior segment model and made significant contributions to our understanding of the mechanisms of outflow and things that might affect it. I saw, and heard, Doug at the American Glaucoma Society meeting in 1995. At that meeting, he did something that I suspect has never been repeated: He played the bagpipes while having his intraocular pressure measured. His intraocular pressure went a long way up, into the 40s, if I recall, so in addition to the damage the bagpipes were doing to our ears, it was potentially affecting his vision as well! Sadly, he died within 2 years from that date.

Doug had a mantra about life and he called it the “Three Es”: Exercise, enthusiasm, and exhilaration. I am not sure which category the bagpipes fell into, possibly all three, but it was like Doug to have enduring passions.

**Fig. 1:**
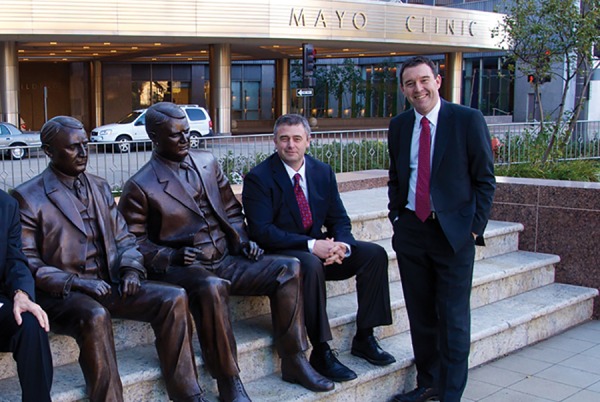
The MAYO clinic

Doug worked at the Mayo Clinic, where he had trained and later became a full professor. He married the two roles of clinician and scientist as well as anyone before or since. And for those of us who try this, it is a difficult road. Not to make matters harder, I do think we need to embrace a further role these days: Administration is a necessary evil, but it should be done by those who understand the “business” -doctors. The Mayo exemplifies this approach and I believe this is one of the factors that gives it the strength that it has (Fig. 1).

I visited the Mayo Clinic when I was the Clinical Director of Ophthalmology at the Royal Victorian Eye and Ear Hospital in Melbourne. I was amazed that the administrator for ophthalmology, who was a lawyer by training, knew of the differences between the anti-VEGFs and the number of the glaucoma procedures that were on offer.

And by all accounts, we have much to do in medicine, and in glaucoma particularly. The lead article in last week's New England Journal of Medicine was by the omnipresent Michael Porter, who is reasonably convinced (and certainly articulate) in regard to how poor doctors are at measuring outcomes. In glaucoma, we may well be reasonably charged with this failing. We focus on easily measured ones, such as intraocular pressures, but fail at the larger issues of “have we actually improved the quality of life for the patient?”

**Fig. 2:**
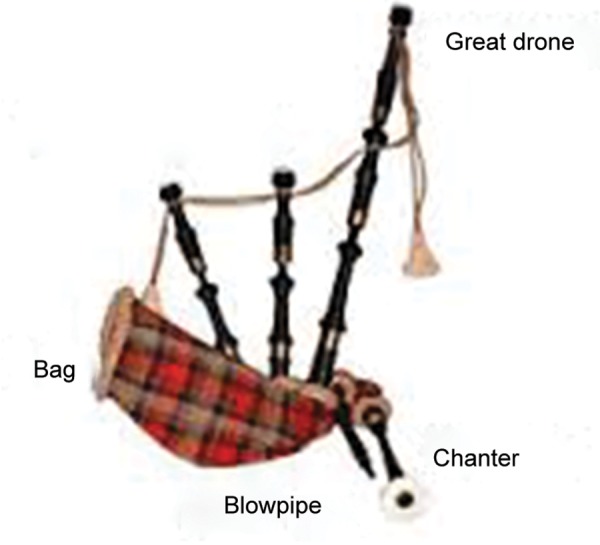
The Bagpipes - note the 'drone'

But, back to the bagpipes. For those who do not know, they are an unusual musical (and I use that term in the loosest possible way) instrument. The note they produce is continuous, without interruption. Some musical punctuation is possible, but mostly it just goes on and on and on. In fact, the main method of altering pitch is called a “drone,” so there you have it (Fig. 2).

## 1995

I finished my glaucoma fellowship in 1995. If you say it quickly, it does not sound so long ago. Quite a few important things happened in 1995. South Africa won the World Cup, eBay started, Toy Story was released, and so was Hugh Grant.

**Fig. 3:**
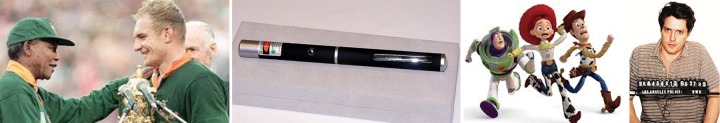
Happenings of 1995

There were a number of important papers released in 1995 and I find it interesting that many of the questions posed by these publications remain with us today (Gandolfi 1995^1^, Smith and Galanis 1995^2^, Hitchings et al 1995^3^) (Fig. 3).

In 1999, at the American Glaucoma Society meeting, I presented a paper called “Microstents in Glaucoma Surgery.” At that time, I thought I had almost cured glaucoma: The impetuousness of youth! I used an existing microperforated tube and inserted them in rabbits and they worked, although, for all sorts of reasons, could not be translated into useful longer-term trials. After presenting the work, it is nice to see some things perhaps have been influenced by that information. But the story of implants is not over and I am sure the current round of glaucoma implants does not solve all our problems either.

My 20-year-old pre-med son has a T-shirt that reads, “I think you will find it is more complicated than that,” which is a quote from Ben Goldacre, a marvelous physician/writer from the UK.

## WHAT TRUTHS DO I NOW KNOW?

So, after 21 years, I am older and apparently wiser and have learned some things. Peter Thiel, the fellow who started PayPal and was the first investor in Facebook, has written a book called *Zero to One.* In this book, he poses the question that he feels all prospective employers should ask of their employees: “What truth do you know that others do not know?” So, as we are working together, here are my answers.

**Graph 1:**
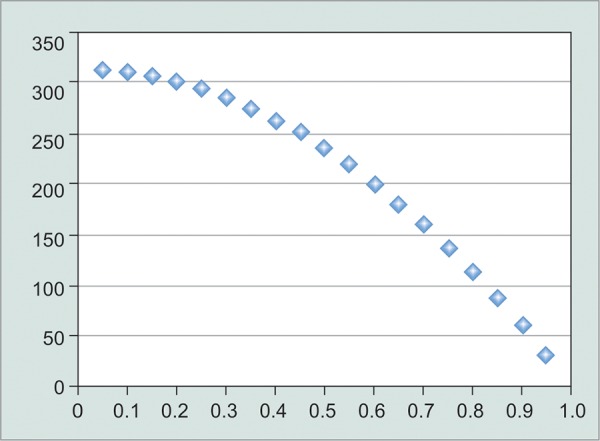
CDR *vs* NRR area

**Graph 2:**
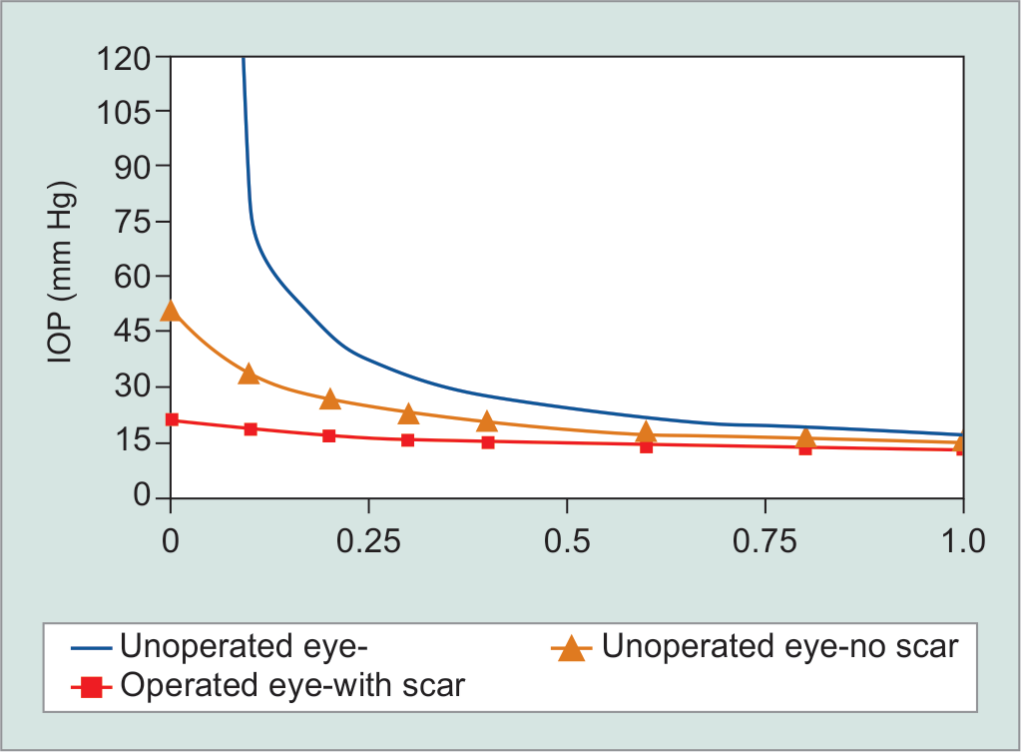
Fraction of normal outflow facility

- *Glaucoma is too expensive to treat:* It affects the patient and relatives in terms of disease burden and quality of life, but the direct costs are staggering. We need to be mindful of costs when we are planning what we do. We have a lot more revisits to surgery, postoperative visits, and it costs a lot more to manage our patients, as we do not tend to cure them. Health service planners look upon us poorly.
- *All the clinical data that we have about a patient's glaucoma is flawed:* What we want to know is outflow resistance and rate of structural and functional change. More than that, we want to be able to predict these things into the future. We know that when clinical trials in glaucoma are performed, they accumulate much more data through more intensive visits and more intensive investigation, usually at a level, i.e., incompatible with practical clinical practice. Most of what clinicians have to deal with would not pass for a clinical trial. We have to treat patients on limited and flawed data.
- *Glaucoma is not a linear disease:* I think intuitively we tend to see linear relationships. A rate of progression that keeps going at the same rate, a risk, i.e., unchanging over time. But glaucoma seems to be full of nonlinear relationships. For example, the neuroretinal rim area to the cup-disk ratio, the intraocular pressure to the outflow facility, visual field scales are nonlinear, and rates of progression are probably nonlinear, certainly from a functional point of view.
  For example, the outflow graph needs a little more thought. I think the implications of this graph are not understood by many eye doctors. If we look at the elevation of intraocular pressure associated with outflow, we see only a modest intraocular pressure elevation when we have about 50% of trabecular meshwork function lost. But if lose another 25% of trabecular meshwork function, the intraocular pressure skyrockets. This piece of information explains why vitreoretinal surgeons call up glaucoma doctors saying the intraocular pressure was fine a week ago (meaning the intraocular pressure was 24 mm Hg on two medicines), but cannot understand why it is now 45 mm Hg. There has only been a small change in outflow facility for that to happen. It also explains why pharma and surgical studies all want higher starting pressures, because the amount of change in outflow to produce significant falls in intraocular pressure is much less (Graphs 1 and 2).
- *Aqueous has to go somewhere:* In the beginning of nonpenetrating glaucoma surgery, the whole process was marred by discussions of aqueous disappearing by nonstandard means, from lakes within the sclera to reabsorption by the choroid. In the end, most of us accept that it filters into the subconjunctival space and is absorbed from there, albeit in a more controlled process. Aqueous that enters the Schlemm's canal needs to get out, and many studies have shown that outflow from Schlemm's canal is reduced in glaucoma.

## WICKED PROBLEMS

Some problems are so complex that you have to be highly intelligent and well informed just to be undecided about them.

―Laurence J Peter

My own research over the past 20 years or so has always delved into the problems of glaucoma surgery, and the more I thought about it, the less I thought I knew for certain. What I can tell you is that this problem has required a lot of tenacity, and perhaps a little intelligence!

A few years ago, we spent 2 years with computational engineers, who were experienced in biologic modeling and porosity, to come to some decision about what was happening in glaucoma surgery and what we needed to be looking at. They spent some time defining the initial conditions, and then later defining the purpose of a glaucoma operation, which they resolved into three key attributes:

1. Aqueous must first leave the eye.
2. It must distribute through the subconjunctival space.
3. It must be absorbed into the vasculature of that space.

When we did more detailed modeling of the bleb and outflow generally,^4^ we came up with some very interesting predications. For example, as the intraocular pressure rises, the bleb appears more avascular. This is because the tissue pressure exceeds capillary driving pressure and blanches vessels. Unfortunately, ischemia tends to drive inflammation, so elevated intraocular pressure and intrableb pressure tends to produce outcomes likely to endanger the bleb function further.

Even our treasured microcyst is not necessarily a good thing. Microcysts represent pockets of extracellular fluid large enough to be visible. Unfortunately, they also represent metabolic isolation of cells, and hence cellular stress and possibly change in the subconjunctival tissue.

**Fig. 4:**
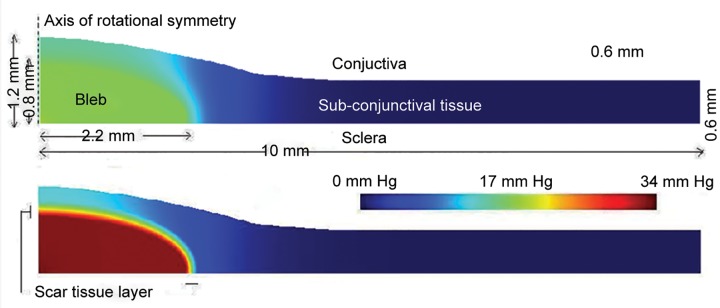
Tissue porosity, lateral porosity through the subconjunctival tissues

What the modeling eventually resolved is that tissue porosity in the subconjunctival space is the key. We do not normally get significant problems with egress and neither with absorption, although both are possible. Tissue porosity, lateral porosity through the subconjunc-tival tissues, is what most often determines intraocular pressure following a surgery (Fig. 4).

To put it another way, hydraulic conductivity of the perisclerostomy subconjunctival tissue is the key. If you model this, you can actual work out how porous the tissue needs to be in order to achieve the intraocular pressure that you want, but beware, calculations are complex and interdependent. Many of our models have failed to take into account the dynamic issues of aqueous production and flow through tissues, and what steady conditions are required for continuous flow.

## NEW ENDPOINTS FOR GLAUCOMA SURGERY

Most glaucoma surgery research has used histology or intraocular pressure as endpoints. The obvious problem is that the sole purpose of glaucoma surgery is to increase facility of the eye, and, i.e., not being tested. Furthermore, because we are getting onto the flatter part of the curve of intraocular pressure and outflow, intraocular pressure is not a very sensitive method of detecting meaningful changes in glaucoma surgery effectiveness.

So, in short, we needed a new endpoint and we needed something that was much more sensitive. We needed to isolate surgical outflow from innate outflow. The porosity of the surgical outflow seemed the most obvious place to start and our modelling data had suggested was the most important aspect.

So we developed a new surgical model for glaucoma.^5^ We took an existing implant, a pediatric Molteno implant, which feels rather like it has been designed for New Zealand white rabbits, although Tony Molteno denies he treats New Zealand rabbits with it! We implanted it in one eye and we tested the outflow facility by direct cannulation of the tube.

We used a glaucoma drainage device because it defined the area of the bleb and had a tube in it, which we could cannulate.

It was not particularly easy to cannulate the tube *in situ,* but it was possible, and it meant that we could identify the function of the implant quite accurately. It was a difficult surgery and the results were as expected.

The model worked very well, but the implant worked really badly to lower pressure. In fact, it was a very consistent outcome. All of the pediatric Molteno implants in New Zealand white rabbits failed, pretty much completely, and certainly to a point where there was a useless level of outflow. It is a very good model of failure and it helped us to measure and correlate histology with porosity.

It is not so hard to tell that the bleb wall on the right-hand side is impervious to fluid. Its porosity is low. Its hydraulic conductivity is low. It will not contribute to surgical outflow, and hence it will not help the intraocular pressure.

Interestingly, when we tested the whole outflow, i.e., glaucoma drainage device + trabecular meshwork in normal rabbits not subjected to a steroid postoperatively, the total outflow was consistently less than prior to surgery. Not by much, but, in short, it meant that a failed tube in a normal rabbit eye left the eye with less outflow than when it started.

## WHAT DETERMINES TISSUE POROSITY?

**Fig. 5:**
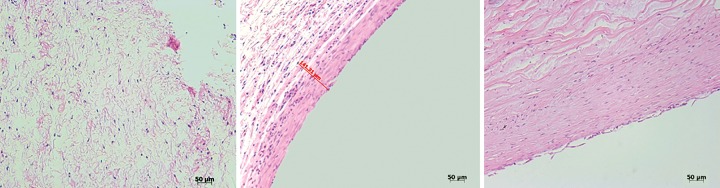
H&E staining of bleb capsule - (left to right) normal conjunctiva, capsule at 4 weeks with no flow, capsule at 4 weeks with aqueous flow

We needed to consider what contributes to the porosity of tissue. Looking at H&E stains, the clear suggestion is the thicker the capsule, the more impervious it is. But on closer inspection there seems to be some exceptions to this rule of thumb (Fig. 5).

**Graph 3:**
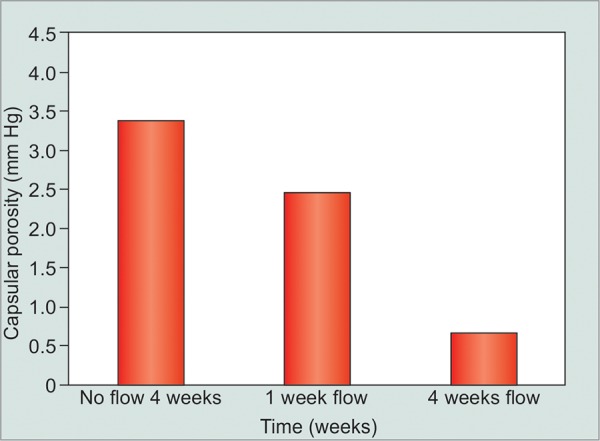
CERA Device outflow directly measured

In very basic terms, tissue porosity appears to be mostly due to extracellular components, collagen, and extracellular matrix. Although collagen contributes to porosity, in the end, it appears that glycosaminoglycans and extracellular matrix play the most important role. It has not been easy to determine this, but glycosamino-glycans, including thrombospondin, has been a material that investigators, such as Tony Molteno have found consistently lining the inside of blebs.

The problem is that when we clinically treat blebs and manage them, we are often managing them to the appearance of cellular activity, i.e., hypervascularity and contracture.

It is not to say that inflammation and wound healing do not have effects on bleb function, clearly they do. But the process has a number of parts and we had no way to separate the effects of aqueous from the effects of wound healing. We were satisfied that tissue porosity was the correct endpoint, so we set about building a new model.

## “CERA MODEL” (CERA = CENTRE FOR EYE RESEARCH AUSTRALIA)

We redesigned the pediatric Molteno implant so that it had two tubes. We did this by additive 3D printing via a number of prototypes and eventually landed on a particular type and shape, using a biologically stable and compatible polymer.^6^

The tubes were attached to the plate using medical-grade silicone and two stoppers were made for each end.

We made implants of different sizes, which was to simulate different volumes of fluid entering the cavity. A smaller plate therefore had a different volume-surface area ratio. In one iteration of experiments, we set up an implantable pump system to pump balanced salt solution (BSS) rather than aqueous around the implant.

Eventually, we settled on using a picoliter pressure-gated syringe pump, which could give us very accurate information about flow around the tube, and hence the capsule. We could place the tube into the front of the eye or not, but, in any event, we had a separate tube through which we could measure the pressure of the system. In effect, now we could measure the capsule porosity exactly and alter the operating conditions of the implant. But we also had histology in the context of porosity data, so for the first time, we could marry structure and function.

The first question we asked is, “are CERA implants comparable to Molteno implants, the plate of which is made out of polypropylene?” The answer appears to be yes. With a different material, but a very similar size and shape, and the porosity and histologic reaction is similar, with no flow around the plate, with 1 week of flow, and with 4 weeks of aqueous flow. Perhaps, this should not surprise as much, as the implant itself is not actually in contact with tissue once it is bathed in aqueous.

Our results mirrored our previous results, which is that glaucoma implants work very well as long as they do not have aqueous running into them. In other words, the porosity of the bleb capsule is high when there has been no fluid flowing through it. The foreign body reaction/wound healing process on its own does not create an impervious scar.

## EFFECTS OF FLUID CHALLENGE

We wondered how stable the porosity was around the plate if there had been no aqueous around it. So we took CERA glaucoma drainage devices, placed them in the eye, but isolated them from the anterior chamber. After 4 weeks, the capsular porosity was measured, and predictably it was good. Capsular porosity was measured at around 12 mm Hg for less than 30 minutes with BSS only (Graph 3).

We then retested the capsule porosity in 3 days. What happened surprised us. Bleb capsule porosity collapsed. The porosity of the bleb appeared to fall by around 80%.^7^ Remember this is with BSS and not with aqueous and it is at a physiologic pressure level. So it seems that you do not need some odd component of aqueous to cause a bleb to fail - BSS will do.

Why does this happen, and why so quickly? Although I cannot answer that at this stage entirely, what I can tell you is that it does appear to occur without a lot of cell ingrowth or histologic change, and without a significant change to the amount of collagen present.

What seems to have happened is that we have a model of failure, which divides the processes into two components. One is classical wound healing, and the other is hydraulic capsular stress.

## WHERE TO FROM HERE?

We are continuously evolving our understanding of glaucoma surgery and we are continually getting better endpoints and methods of manipulation. Subconjunctival drainage is still the most effective and reliable system that we have, and it works much of the time. Our problems stem from not knowing well enough why it does not work in some people.

## FINAL THOUGHTS

And I will leave you with some provocations, perhaps to focus us for the next 21 years, although I shall be over 70 at that stage and I am not sure I will be contributing substantially to the debate!

- How much should 1 mm Hg fall in intraocular pressure (IOP) for a patient cost?
  As I started this talk, glaucoma treatment is expensive and we will need to put some relative value to our interventions.^8^ It is, of course, more complicated than actually how much it costs, as we have to take into account safety, longevity, and patient preference. But at some point, we will have to know whether something is as valuable as another and how much price we are paying for mild improvement in safety and efficacy.
- We should be measuring outflow, as it is the problem. It is true that patients have differing sensitivity to intraocular pressure, but, in essence, glaucoma is an intraocular pressure-dependent disease and elevated intraocular pressure occurs because of reduced outflow. The fact that we still do not regularly measure outflow, before or after surgery, seems to be a significant lack.
- We should measure optic nerve health; after all, it is the patient's problem.
  We are all aware that patients have differing sensitivity to pressure and having some method of measuring optic nerve stress with accuracy would be a significant contribution. Distress of the optic nerve is the patient's main risk.
- We should consider all of glaucoma management as risk management.
  Glaucoma does not (usually) produce symptoms in and of itself, so we are really only trying to produce a meaningful reduction in risk of visual loss as a result of intervention. Given that all forms of intervention produce risk, our question should be “how much risk management do we achieve for this amount of cost?”

## References

[1] Gandolfi SA (1995). Improvement of visual field indices after surgical reduction of intraocular pressure.. Ophthalmic Surg.

[2] Smith SG, Galanis JC (1995). One-year results of the intrascleral glaucoma implant.. J Cataract Refract Surg.

[3] Hitchings RA, Wu J, Poinoosawmy D, McNaught A (1995). Surgery for normal tension glaucoma.. Br J Ophthalmol.

[4] Gardiner BS, Smith DW, Coote M, Crowston JG (2010). Computational modelling of fluid flow and intra-ocular pressure following glaucoma surgery.. PLoS One.

[5] Nguyen DQ, Ross CM, Li YQ, Pandav S, Gardiner B, Smith D, How AC, Crowston JG, Coote MA (2012). A model to measure fluid outflow in rabbit capsules post glaucoma implant surgery.. Invest Ophthalmol Vis Sci.

[6] Ross C, Pandav SS, Li YQ, Nguyen DQ, Beirne S, Wallace GG, Shaarawy T, Crowston JG, Coote M (2015). Determination of bleb capsule porosity with an experimental glaucoma drainage device and measurement system.. JAMA Ophthalmol.

[7] Pandav SS, Ross CR, Faisal TT, Nada R, Singh N, Gautam N, Beirne S, Wallace G, Sherwood M, Crowston JG Porosity of bleb capsule declines rapidly with fluid challenge.. J Curr Glaucoma Pract.

[8] Coote M (2013). Stenting eyes: the pressure to perform.. Clin Experiment Ophthalmol.

